# New Insights into the Role of Secondary Metabolic Pathways in Resistance of Potato to *Dickeya solani*

**DOI:** 10.3390/ijms26178370

**Published:** 2025-08-28

**Authors:** Anna Grupa-Urbańska, Katarzyna Szajko, Waldemar Marczewski, Renata Lebecka

**Affiliations:** Plant Breeding and Acclimatization Institute–National Research Institute, Radzików, Młochów Division, Platanowa Str. 19, 05-831 Młochów, Poland; k.szajko@ihar.edu.pl (K.S.); w.marczewski@ihar.edu.pl (W.M.)

**Keywords:** *Dickeya solani*, potato resistance, transcriptome profiling, KEGG analysis, pathogenesis pathways, *POPA* gene

## Abstract

*Dickeya solani* causes soft rot in potato (*Solanum tuberosum* L.) tubers. We used bulk RNA-seq to compare the early transcriptional responses of the diploid F_1_ genotypes from the mapping population that varied in tuber resistance to *D. solani*. RNA was collected from wounded tubers inoculated with *D. solani* (B), wounded tubers treated with sterile water (W), and non-treated tubers (NT) at 8, 24, and 48 hours post-inoculation (hpi). The largest transcriptional divergence between resistant (R) and susceptible (S) genotypes occurred at 8 hpi, with R tubers showing stronger induction of phenylpropanoid biosynthesis, phenylalanine and tyrosine metabolism, amino sugar and nucleotide sugar metabolism, isoquinoline alkaloid biosynthesis, and glutathione metabolism. Phenylpropanoid biosynthesis was dominant in R tubers, in 17 differentially expressed genes (DEGs), consistent with rapid suberin and lignin deposition as a physical barrier. RT-qPCR of nine defence-related genes corroborated the RNA-seq trends. The suberisation-associated anionic peroxidase *POPA* was located within a QTL for *D. solani* resistance on chromosome II, supporting its role as a candidate for future functional studies. This is the first transcriptome-based comparison of R and S potato genotypes challenged with *D. solani*, providing candidate pathways and genes that may guide future molecular breeding once their roles are validated.

## 1. Introduction

Potato (*Solanum tuberosum* L.) is an essential crop species for global food security, ranking as the fourth most widely consumed staple food worldwide, after rice, wheat, and maize [1,2]. The yield of potato crops is under threat from bacterial pathogens, including *Dickeya solani*, a highly aggressive member of the *Pectobacteriaceae* family. *D. solani* and other pectinolytic bacteria are among the top ten most damaging plant pathogenic bacteria globally [3]. In potato, this pathogen is responsible for blackleg and soft rot, which cause significant pre- and post-harvest losses. It has been demonstrated that even minimal inoculum levels of *D. solani* are capable of initiating the infection process [4,5].

Resistance to *D. solani* in potato is a polygenic trait [6]. It has been demonstrated that diploid hybrids derived from crosses between *S. tuberosum* and wild *Solanum* species exhibit significantly higher resistance to bacterial pathogens compared to cultivated potato, thus offering a promising source for resistance breeding programmes [7,8]. Two of the highly resistant interspecific hybrid clones were utilised in order to locate quantitative trait loci (QTLs) for tuber and leaf resistance to *Pectobacterium atrosepticum* (formerly *Erwinia carotovora* ssp. *atroseptica* (van Hall) Dye) [6], and for tuber resistance to *D. solani* on the potato molecular map [9]. The high level of tuber resistance in diploid hybrids is manifested either by the complete inhibition of bacterial growth via suberin formation or by slower potato tissue maceration than in susceptible clones. Therefore, two separate parameters were used to characterise the resistance of potato tubers to bacteria: disease incidence (DI) and disease severity (DS). DI was calculated as the ratio between the number of tubers exhibiting symptoms of rot to the total number of tubers that were tested. DS was calculated as the mean mass of macerated tissue only from tubers exhibiting symptoms of infection. These parameters were utilised in our previous studies, in a comparative analysis of differently expressed proteins induced at the early phase of *D. solani* infection and in QTL mapping [9,10]. Two significant QTLs for resistance to *D. solani* were mapped on chromosomes II and IV in the biparental segregating population DS-13 [9]. The QTL on chromosome IV demonstrated a strong correlation with reduced tissue decay and a lower proportion of infected tubers, accounting for up to 22.9% of the phenotypic variance. Similarly, the QTL on chromosome II primarily influenced the severity of tissue damage, accounting for 16.5% of the observed variance [9].

Pathogen infection can initiate alterations in the expression of a variety of metabolic pathways. The phenylpropanoid pathway is responsible for the production of numerous secondary metabolites, including lignin and suberin. In potato tubers, suberisation processes are induced by wounding, which is followed by the formation of protective barriers to prevent or significantly delay the onset of tissue decay. Suberisation involves the deposition of phenolic and aliphatic suberin components, forming a physical and chemical barrier to infection and water loss [11,12]. Recent studies have revealed a temporal regulation of suberin biosynthesis, with phenolic components being deposited earlier during the wound-healing process, followed by the aliphatic components that strengthen the protective barrier [12]. The process of suberisation, which is rapid and effective, plays a crucial role in preventing the spread of pathogens within infected tubers. The infection process consists of two distinct phases: an initial asymptomatic biotrophic phase, during which bacteria silently proliferate within host tissues without causing visible symptoms, and a symptomatic necrotrophic phase characterised by the expression of plant cell wall-degrading enzymes [13,14]. The transition between these phases is determined by a number of environmental factors, including the availability of oxygen, temperature, and nutrient conditions [15].

A comparison of gene expression profiles between pathogen-inoculated and mock-inoculated plants provides information on how plants respond to a pathogen attack, rather than how they resist infection. In order to identify genes that may be involved in resistance processes, a comparative analysis of plants exhibiting contrasting levels of resistance should be performed [16]. For complex traits, the employment of bulk sample analysis with biparental populations can facilitate the selection of extreme individuals. There are two approaches to studying plants characterised by extremely different phenotypes in terms of polygenic traits. Initially, tissues are sampled from the phenotypic extremes. Then, a single DNA/RNA/protein analysis is performed. Alternatively, the DNA/RNA/protein is isolated first from a selected set of individuals, and then, plants with extreme molecular patterns are evaluated for their phenotypic variation [17]. Here, we used a combination of bulk RNA sequencing and comprehensive bioinformatic analysis to identify key metabolic pathways that are potentially involved in potato resistance to *D. solani*. We hypothesise that a high level of resistance to *D. solani* in potato tubers is the result of molecular events at an early stage of infection. Our earlier research [10] indicates that 8 hpi corresponds to the early stage of infection, when symptoms of rot are already visible. Here, we showed for the first time differences in the activity of molecular pathways in potato tubers, comparing highly resistant and susceptible potato genotypes. The genes selected at the beginning of the infection process may be key to the understanding of the response of highly resistant potato genotypes to bacteria.

## 2. Results

### 2.1. Phenotype of Selected Genotypes

**Figure 1:**
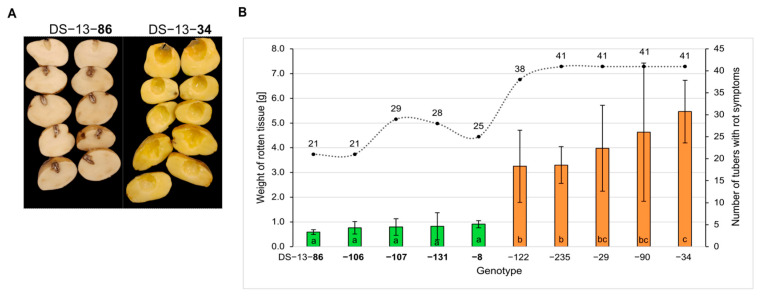
Phenotypic assessment of potato tubers infected with *D. solani*, according to method described by Lebecka et al. [9]. (**A**) Representative images of tubers from the resistant genotype DS-13-86 (left) and the susceptible genotype DS-13-34 (right). Rotten tissue was removed before photography. (**B**) Phenotypic assessment of 5 resistant (green) and 5 susceptible (orange) potato genotypes that were obtained in seven independent experiments, 41 tubers per genotype, from four growing seasons; mean weight of rotten tissue of tubers with symptoms of infection; the dashed line represents the number of tubers with rot symptoms (disease incidence, DI); means followed by the same letter are not significantly different at *p* ≤ 0.05 according to Duncan’s test. (**B**) Error bars indicate the standard deviations.

Three days after wound-inoculation with *D. solani*, potato tubers exhibited two types of reactions: symptoms of wet macerated tissue or dry necrotic and suberised lesions (Figure 1A). An evaluation of tuber resistance to *D. solani* revealed significant differences between resistant and susceptible potato genotypes (*p* < 0.01) (Figure 1B). The disease severity (DS), measured as the mean weight of macerated tissue, was found to be significantly lower in resistant genotypes compared to susceptible ones. The mean DS in resistant genotypes was 0.78 g, ranging from 0.59 to 0.91 g. These genotypes exhibited limited tissue maceration, which is often associated with the formation of necrotic, suberised wound zones. The mean number of tubers exhibiting rot symptoms in resistant genotypes was 24.8 out of 41 tested (DI = 0.60), ranging from 21 to 29. The remaining tubers exhibited symptoms of dry necrotic and suberised lesions. In contrast, the mean DS in susceptible genotypes was 4.13 g, with a range from 3.25 to 5.46 g. Furthermore, a higher proportion of tubers exhibited symptoms of rot. The mean number of tubers exhibiting symptoms of rot in susceptible genotypes was found to be 40.4 out of 41 tested (DI = 0.98).

### 2.2. Sequencing Data

Next-generation sequencing was performed on 54 RNA samples from resistant and susceptible potato genotype bulks subjected to three treatments (inoculated, wounded control, untreated control) at three time points (8, 24, and 48 hpi). Libraries generated 10–31 million raw reads per sample. All raw reads are available under BioProject PRJNA1264659, and the sequencing depth per sample was in average 28.8 M. Differential expression was analysed in DESeq2 using an FDR-adjusted *p* ≤ 0.01 (Benjamini–Hochberg) and log_2_ fold change ≥ 2, comparing resistant vs. susceptible bulks (BS vs. BR, WS vs. WR, and NTS vs. NTR) at each time point. On average, 21,215 genes were differentially expressed, including a mean of 3021 significantly upregulated genes (Table 1).

### 2.3. Principal Component Analysis (PCA)

**Figure 2:**
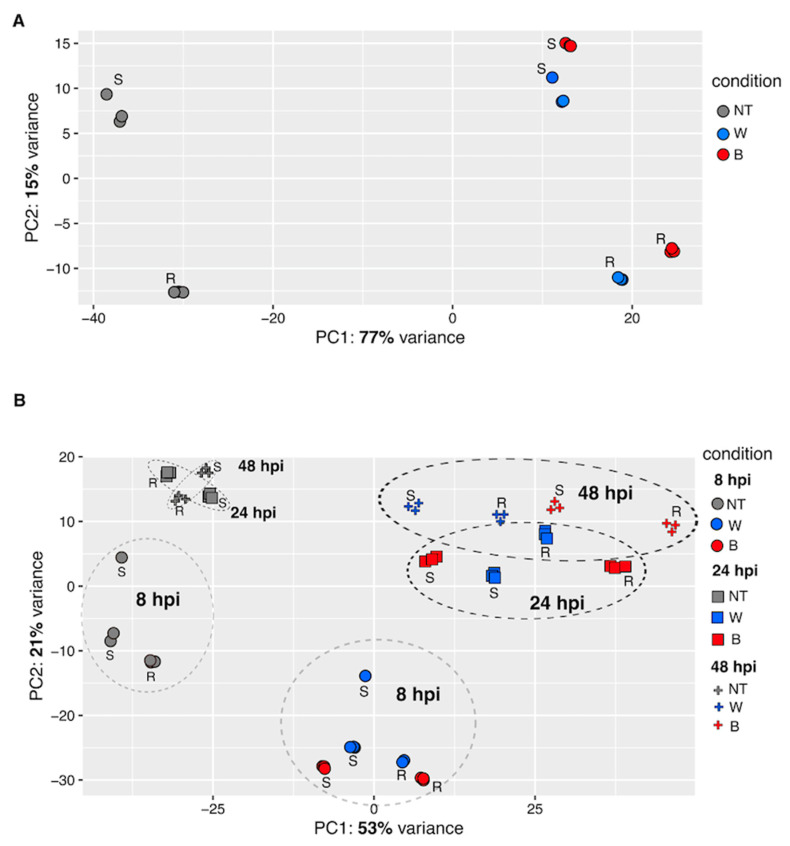
PCA of transcriptome response to wounding and *D. solani* infection in potato tubers differing in resistance. (**A**) PCA plot showing global gene expression variation at 8 hours post-inoculation (hpi). Samples are coloured according to treatment: not-treated (NT, grey), wounded and water-treated (W, blue), and *D. solani*-inoculated (B, red). Genotypes are indicated as resistant (R) or susceptible (S). (**B**) PCA of all time points: 8 hpi (circles), 24 hpi (squares), and 48 hpi (crosses). Samples are shaped by time point and coloured by treatment.

Principal Component Analysis (PCA) was performed using normalised gene expression values on data after VST transformation (variance stabilising transformation, DESeq2) to assess global transcription in potato tubers in response to wounding and *D. solani* infection across time points and genotypes differing in resistance (Figure 2). At 8 hpi, PCA (Figure 2A) showed that the first two principal components (PC1 and PC2) accounted for 92% of the total variance in gene expression (PC1: 77%, PC2: 15%). Samples grouped according to treatment include non-treated (NT), wounded and water-treated (W), and *D. solani*-inoculated (B) groups. The *D. solani*-inoculated samples formed a separate cluster along PC1, indicating a strong transcriptional response to bacterial infection. Genotypic differences were also evident, with resistant (R) and susceptible (S) genotypes showing distinct spatial separation within treatment groups. In the time course PCA (Figure 2B), PC1 and PC2 explained 74% of the variance (PC1: 53%, PC2: 21%). Temporal progression was evident, with samples shifting position over time, particularly in the *D. solani*-infected group. Elliptical clustering indicated consistent transcriptomic changes across time points with the most pronounced divergence observed at 48 hpi. Notably, samples from resistant and susceptible genotypes exhibited distinct clustering patterns following bacterial inoculation.

### 2.4. Detection of DEGs

**Figure 3:**
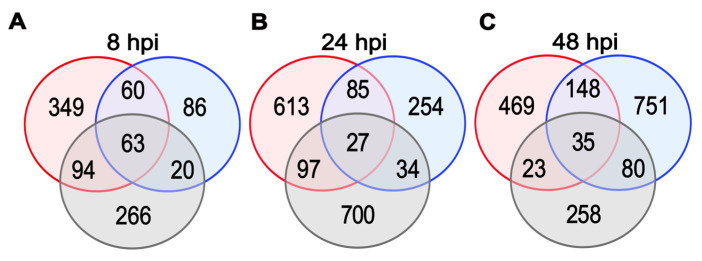
Venn diagrams showing the overlap of significantly upregulated DEGs at 8 (**A**), 24 (**B**) and 48 (**C**) hours post-inoculation (hpi) in BS vs. BR (red), WS vs. WR (blue), and NTS vs. NTR (grey) comparisons.

DEGs between resistant and susceptible samples, across the three treatments, B (red colour), W (blue colour), and N (grey colour), at 8, 24, and 48 hpi, selected based on the applied threshold *p* ≤ 0.01, log_2_ fold change ≥ 2, are shown in Figure 3. Each circle represents the set of DEGs that are specific to one treatment, while overlapping regions indicate genes commonly regulated across two treatments. The intersection of all three circles represents DEGs shared across all treatments. At 8 hpi, 349 upregulated DEGs were detected only in B, compared to 266 in NT and 86 in W. Sixty DEGs were shared between B and W. At 24 hpi, the number of DEGs in B increased to 613, while W and NT had 254 and 700 upregulated DEGs, respectively. Eighty-five DEGs overlapped between B and W, and twenty-seven were common to all three conditions. At 48 hpi, B samples contained 469 DEGs, while W and NT had 751 and 258 DEGs, respectively. Seventy-four DEGs were uniquely upregulated in infected tubers at this time point.

**Figure 4:**
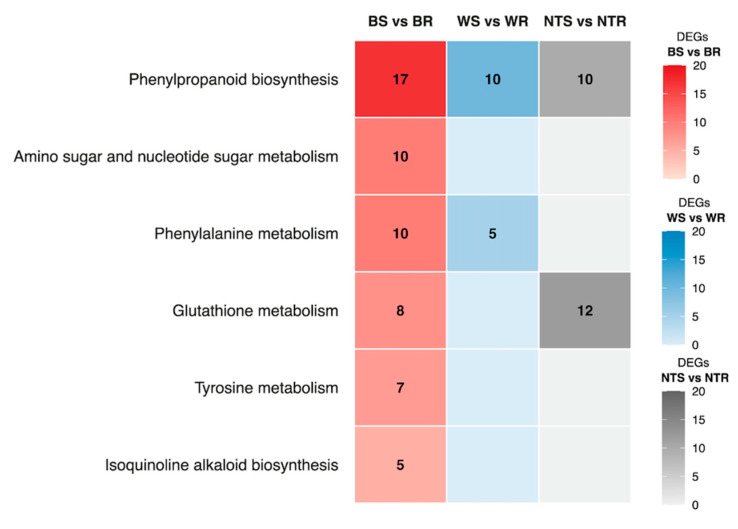
Heatmap showing the six most enriched KEGG pathways among the upregulated genes 8 hours post-inoculation (hpi). Each tile shows the number of differentially expressed genes (DEGs) for one of three contrasts: BS vs. BR (red scale), WS vs. WR (blue), and NTS vs. NTR (grey).

A total of 566 DEGs were identified as being significantly upregulated between the BR and BS bulks at 8 hpi. They included the following metabolic pathways: ‘phenylpropanoid biosynthesis’, ‘phenylalanine metabolism’, ‘tyrosine metabolism’, ‘isoquinoline alkaloid biosynthesis’, ‘amino sugar and nucleotide sugar metabolism’ and ‘glutathione metabolism’. The phenylpropanoid biosynthesis category was the most numerous, including 17 DEGs. Two sets of genes with a common function were detected: three DEGs, LOC102582618, LOC102604380, LOC102577727 were associated with two biological pathways, and three DEGs, LOC102583802, LOC102600413, LOC102581835, were associated with three KEGGs pathways (Table 2). Of the 45 DEGs selected at 8 hpi, 15 and 17 were also upregulated at 24 and 48 hpi, respectively. We also used DEGs for WS vs. WR and NTS vs. NTR comparisons to identify KEGG pathways. The pathways that overlap with the pathways identified in B treatment are shown in Figure 4.

Five genes were detected, for which the expression levels were significantly upregulated at three stages after *D. solani* infection. However, there was no upregulation observed in the W treatment group. There were LOC102599828, LOC102577694, LOC102584603, LOC107059944, LOC102583127 genes, which were functionally related to lignin-forming anionic peroxidase-like, suberisation-associated anionic peroxidase, 4-coumarate-CoA ligase 2, chitotriosidase-1-like, and endochitinase EP3, respectively (Table 2).

Of the 566 DEGs identified in the BR vs. BS comparison at 8 hpi, 18 genes were found to be located within QTL regions on chromosomes II and IV (Table 3), as previously reported for the same trait [9].

### 2.5. RT-qPCR Validation

**Figure 5:**
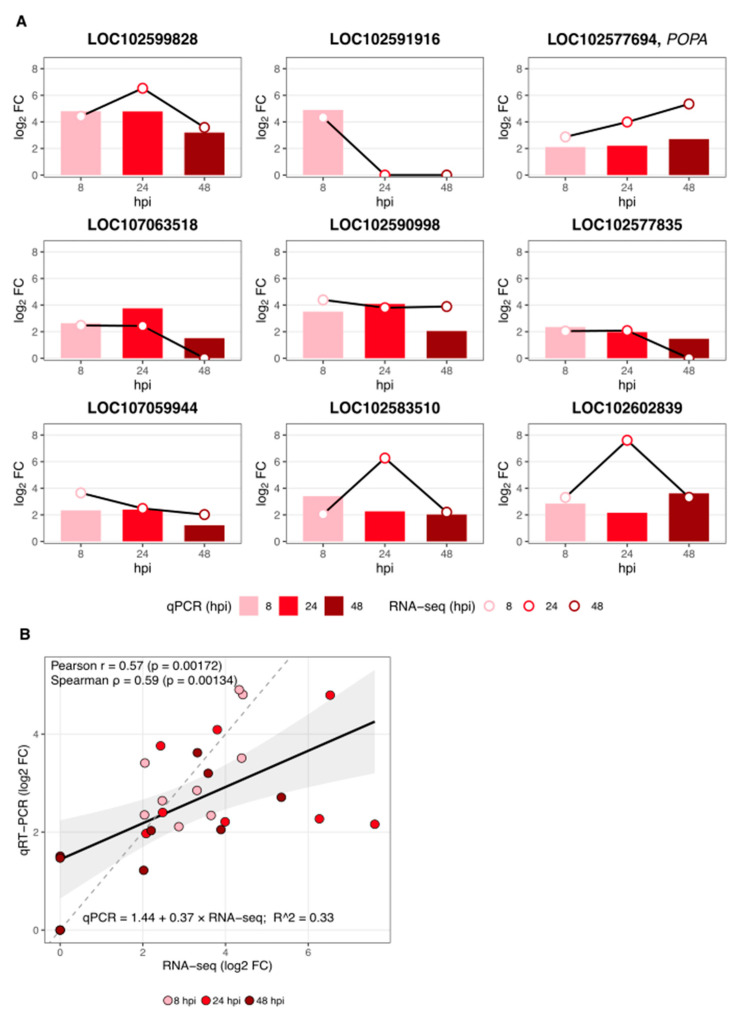
Validation of RNA-seq by RT-qPCR for nine defence-related genes and global concordance between methods. (**A**) For each gene (LOC102599828, LOC102591916, LOC102577694 (*POPA*), LOC107063518, LOC102590998, LOC102577835, LOC107059944, LOC102583510, and LOC102602839), bars show RT-qPCR log_2_ fold change (Resistant vs. Susceptible bulks) at 8, 24, and 48 hours post-inoculation (hpi) in light, medium, and dark red, respectively. The black line with open circles depicts RNA-seq log_2_ fold change at the matching time points. RNA-seq DEGs (|log_2_FC| ≥ 2, *p* ≤ 0.01); open circles denote RNA-seq values. (**B**) Correlation between RNA-seq and RT-qPCR across all measurements (*n* = 27). Points are coloured by time (8, 24, 48 hpi). The solid line shows the least-squares fit with the 95% confidence band; the dashed line indicates the identity line (y = x). Correlation and regression statistics: Pearson r = 0.57 (*p* = 0.00172), Spearman ρ = 0.59 (*p* = 0.00134), and linear model qPCR = 1.44 + 0.37 × RNA-seq (R^2^ = 0.33).

**Figure 6:**
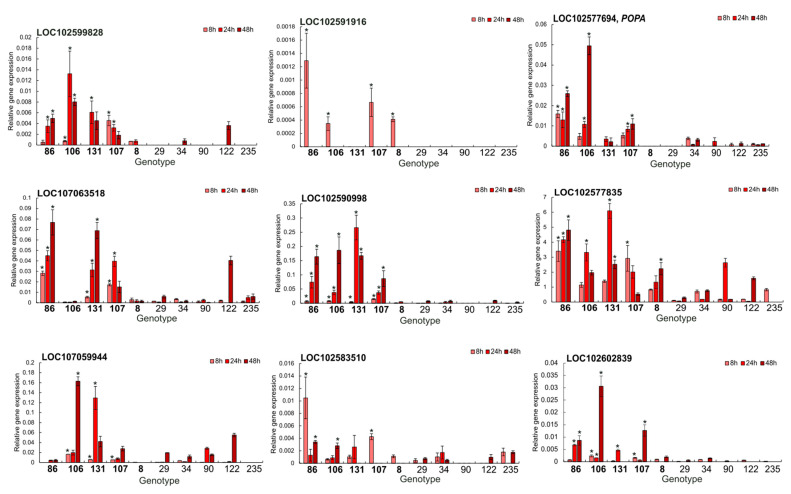
Relative expression of nine defence-related DEGs in five resistant (bold-labelled) and five susceptible (regular-labelled) *F_1_* individuals at 8 (light pink), 24 (medium pink), and 48 (dark pink) hours post-inoculation (hpi) with *D. solani*. Panels correspond to the following genes: LOC102599828, LOC102591916, LOC102577694 (*POPA*, suberisation-associated anionic peroxidase), LOC107063518, LOC102590998, LOC102577835, LOC107059944, LOC102583510, and LOC102602839. Bars represent mean ± SE from three technical replicates for each biological replicate (individual genotype, three independent tubers). Asterisks indicate statistically significant differences (*p* < 0.05, Duncan’s test) between genotypes at the corresponding time point.

To validate the bulk RNA-seq dataset, nine differentially expressed, defence-related genes were selected for RT-qPCR: LOC102599828, LOC102591916, LOC102577694 (*POPA*), LOC107063518, LOC102590998, LOC102577835, LOC107059944, LOC102583510 and LOC102602839. Figure 5A shows, for each gene, the RT-qPCR log_2_ fold change at 8, 24, and 48 hours post-inoculation (hpi) (bars in light/medium/dark red) together with the corresponding RNA-seq values (black line with open circles). Across all gene × time observations (*n* = 27), RNA-seq and RT-qPCR were moderately but significantly correlated (Figure 5B: Pearson r = 0.57, *p* = 0.00172; Spearman ρ = 0.59, *p* = 0.00134). The correlation was strongest at 48 hpi (r = 0.73, *p* = 0.0248), moderate at 8 hpi (r = 0.65, *p* = 0.056), and weaker at 24 hpi (r = 0.46, *p* = 0.207). A simple linear model (qPCR = 1.44 + 0.37 × RNA-seq; R^2^ = 0.33) indicates overall agreement in direction, with a reduced amplitude of fold changes in RT-qPCR relative to RNA-seq. Genotype-level transcript abundances for the five resistant and five susceptible F1 individuals used for bulking at 8, 24, and 48 hpi are shown in Figure 6. In general, resistant genotypes displayed a higher level of expression of the selected genes. However, there were also genotype-specific differences, and the timing of peak expression differed between genes and genotypes. For example, some genes in resistant genotypes were induced early, at 8 hpi, and reached maximal levels at 24 hpi or 48 hpi (LOC102590998, LOC102577835, LOC102603839). LOC102577694 (*POPA*), encoding suberisation-associated anionic peroxidase, was strongly induced in three resistant genotypes with transcript accumulation increasing over time and generally peaking at 48 hpi. LOC102591916, encoding a peroxidase 43-like protein involved in early lignin biosynthesis, was analysed only at 8 hpi, consistent with its RNA-seq-based induction restricted to that time point, and exhibited expression only in four resistant genotypes (Figure 6). These patterns indicate both gene- and genotype-specific timing of defence responses to *D. solani*.

## 3. Discussion

Potato tubers exhibiting contrasting levels of resistance were used for the bulk RNA-seq analysis. Due to environmental conditions significantly influencing the expression of potato tuber resistance to pectinolytic bacteria, the assessment of the resistance of selected genotypes from the DS-13 mapping population [9] was repeated during the RNA-seq experiment. Results from four years of testing tuber resistance to *D. solani* confirmed significant differences in the resistance of tubers from the five most resistant and five most susceptible potato genotypes used in these studies.

The PCA underscore the dynamic and treatment-specific nature of transcriptomic responses in potato tubers following *D. solani* infection. The clear separation of inoculated samples from controls and wounded-only treatments along PC1 at 8 hpi suggests that bacterial infection triggers robust and distinct transcriptional reprogramming early in the interaction. The clustering of resistant and susceptible genotypes within treatment groups further implies the genotype-dependent modulation of defence related pathways. The separation of resistant and susceptible genotypes along PC2 as early as 8 hpi suggests that genotype-specific defence mechanisms are rapidly engaged. Temporal PCA patterns reveal that transcriptomic changes are not static but evolve significantly over time, with increasing divergence from baseline profiles observed at 24 and 48 hpi. This progression likely reflects the activation and resolution of defence mechanisms as well as pathogen-induced stress response. The separation of *D. solani*-inoculated samples across all time points highlights the persistent impact of infection on host gene expression.

There are two main phases of potato tuber infection with pectinolytic bacteria: asymptomatic and symptomatic. Bacteria remain in a latent mode after entering the potato tuber and they start to multiply when conditions are favourable for their growth. They produce small, diffusible molecules. Once a critical concentration of these molecules has been reached, the quorum sensing system activates the expression of genes that encode pectin cell wall-degrading enzymes [19]. To evaluate potato tuber resistance to pectinolytic bacteria, we created favourable conditions for the development of rot symptoms. In our preceding studies, we demonstrated that under specific conditions (i.e., temperature of incubation set at 27 °C, high humidity, and application of the highly aggressive isolate of *D. solani*), the symptoms of infection on potato tubers are not visible after seven hours, but are after eight hours from inoculation [10]. This asymptomatic phase likely reflects the biotrophic-like behaviour of the pathogen during initial invasion, followed by the transition to the necrotrophic phase of infection when an extensive tissue degradation is apparent.

Suberisation is known to proceed in a biphasic manner, beginning with the deposition of phenolic precursors within 24 h post-wounding, followed by aliphatic components that complete the apoplastic barrier [12]. Consistent with this model, the upregulation of genes encoding peroxidases and suberin-related enzymes at 8 hpi suggests that resistant genotypes activate these barriers more rapidly, contributing to the containment of the pathogen before symptom onset. RT-qPCR validation (Figure 5) confirmed the RNA-seq trends across 8, 24, and 48 hpi. Quantitatively, RNA-seq and RT-qPCR were moderately but significantly correlated (Figure 5B; Pearson r = 0.57, *p* = 0.00172; Spearman ρ = 0.59, *p* = 0.00134). The fitted regression (qPCR = 1.44 + 0.37 × RNA-seq; R^2^ = 0.33) indicates a smaller dynamic range for RT-qPCR relative to RNA-seq, in line with cross-platform assessments of these methods [20,21]. Such differences can arise from distinct normalisation strategies and primer-dependent amplification efficiency in qRT-PCR [22]. Despite these quantitative discrepancies, the direction of regulation was consistent across methods, supporting the robustness of the RNA-seq dataset used for pathway-level interpretation. The discrepancies observed may indicate the molecular complexity of the potato’s response to *D. solani* infection.

In resistant plants, amino sugar and nucleotide sugar metabolism plays a crucial role in various aspects of defence and adaptation to stress. It has been established that these pathways are involved in the synthesis of complex carbohydrates, such as cell wall components, and that they play a part in signalling and responding to environmental challenges [23]. Li and Zhang [24] showed that changes in the transcriptome caused by the alteration of a single gene are frequently highly pleiotropic and greatly influenced by the genetic background. Accordingly, we interpret the DEGs identified here in the context of entire metabolic pathways rather than attributing causality to individual genes.

Plant response to stress is a highly complex process that leads to specific modifications at the genetic/epigenetic, cellular, and physiological levels. The phenomenon of the upregulation of gene expression is particularly attributed to plant response to stress conditions that can lead to an intensified synthesis of corresponding proteins. In the present study, we investigated upregulated gene expression in tubers at the initial stage of infection to reveal metabolic pathways whose activity can be particularly important in potato resistance to *D. solani.*

The glutathione metabolism pathway was enriched in DEGs both in inoculated and control samples. This pathway plays an important role in plant response to abiotic stress and biotic stress [25]. For example, in tomato, resistant interactions with *Pseudomonas syringae* pv. *tomato* are associated with higher constitutive and induced glutathione S-transferase activity [26]. In our study, resistant tubers showed a trend towards increased expression of glutathione-related genes after *D. solani* inoculation. While these patterns are consistent with a role in stress adaptation, the evidence is associative, and further experiments will be needed to determine whether glutathione metabolism directly contributes to *D. solani* resistance.

Secondary metabolites (SMs) have diverse biological functions in plants’ reactions to biotic and abiotic stress. Approximately 200,000 SMs have been isolated and characterised in plants [27]. They are generally synthesised through incredibly complex metabolite pathways [28]. In the early stages of infection, the plant synthesises lignin and callose, which serve to restrict the spread of pathogens throughout the plant tissues [29]. We recognised 45 genes involved in the defence response of potato tubers to *D. solani* infection that were associated with six SM pathways. Seventeen DEGs identified in our dataset were annotated to the phenylpropanoid biosynthesis pathway (Table 2). This pathway is known to contribute to the synthesis of compounds such as suberin, lignin, stilbenes, flavonoids, phytoalexins, and coumarins [30,31], many of which have documented roles in plant defence. Suberised cells, deposited in response to wounds, are a protective layer preventing pathogen infection. In our study, we found that five of these proteins (phenylalanine ammonia lyase-like, three 4-coumarate ligases, and cinnamoyl-CoA reductase) are associated with DEGs that play a key role in suberin biosynthesis [32]. We observed a rapid upregulation of these genes in resistant tubers at 8, 24, and 48 hpi following bacterial inoculation, but not after wounding alone. While this association suggests a possible role of the phenylpropanoid pathway in early defence responses, the present data are correlative, and direct functional involvement in *D. solani* resistance remains to be experimentally confirmed. Biosynthesis of lignin proceeds from L-phenylalanine via a complex biochemical pathway which includes the activity of 4-coumarate-CoA ligase [33]. In potato, the anionic peroxidase associated with the suberisation response in tubers during wound healing was characterised [34].

Pathogenesis in potato tubers related to *D. solani* infection is the effect of the interplay between the tuber wounding process and bacterial penetration. Three genes (lignin-forming anionic peroxidase-like, suberisation-associated anionic peroxidase (*POPA*), and 4-coumarate-CoA ligase 2) were consistently upregulated significantly at 8, 24, and 48 h in resistant tubers following *D. solani* inoculation, with no significant change in control treatments. The expression pattern is consistent with a potential role in reinforcing cell walls and suberisation during infection. However, given that our study is based on transcriptomic data, these observations should be interpreted as correlations. Functional studies, such as gene silencing or overexpression, will be required to establish a causal link between their expression and the observed resistance phenotype. In studies of Du et al. [35] and Yang et al. [36], the expression of the *StPOPA* gene (suberisation-associated anionic peroxidase) in potato plants was found to increase resistance to *Phytophthora infestans* infection. This was achieved by increasing the accumulation of callose in the cell walls and reactive oxygen species (ROS), which limited the spread of the pathogen, likely through programmed cell death [36].

In our previous paper, we mapped two significant QTLs for resistance to *D. solani* on chromosomes II and IV [9]. In this study, of the 566 upregulated DEGs between BS and BR at 8 hpi, 18 were localised in these QTLs (Table 3). Among these, the locus of the gene suberisation-associated anionic gene *POPA* (LOC102577694) was identified on potato chromosome II within a quantitative trait locus (QTL) for resistance to *D. solani,* suggesting a possible link, although based on corelative transcriptomic evidence, it can be considered as the main candidate gene.

## 4. Materials and Methods

### 4.1. Plant Material

The plant material used in this study consisted of tubers from individuals selected from the progeny of the DS-13 mapping population. The development of this population was achieved through the cross-breeding of two diploid potato (*Solanum tuberosum* L.) clones derived from the Młochów collection of diploid potato plants [8]: one that is highly resistant to *D. solani*, DG 00–270, and one that is highly susceptible, DG 08–305. Progeny clones with contrasting levels of resistance to infection by *D. solani* were selected on the basis of previous research [9]. These included five clones with higher levels of resistance, DS-13-8, DS-13-86, DS-13-106, DS-13-107, DS-13-131, and five clones with lower levels of resistance, DS-13-29, DS-13-34, DS-13-90, DS-13-122, DS-13-235.

### 4.2. Bacterial Inoculum

The *Dickeya solani* strain IFB0099, provided by Professor E. Lojkowska (University of Gdańsk, Gdańsk, Poland), was used in this study. Its draft genome is available in GenBank (assembly GCA_000831935.1, accession JXRS00000000) and was first reported by [37]. The strain is also maintained in the Plant Research International collection, Wageningen, The Netherlands (syn. IPO2276). The strain was stored at −70 °C, grown on Luria–Bertani (LB) agar (Sigma-Aldrich, St. Louis, MO, USA) at 27 °C for 24 h, and resuspended in sterile deionised water. The suspension was adjusted to optical density OD_600_ = 1.0 (10^9^ colony-forming units (CFU) mL^−1^) using a Hitachi U-1900 spectrophotometer (Hitachi High-Tech Corporation, Tokyo, Japan).

### 4.3. Experimental Procedure

**Figure 7:**
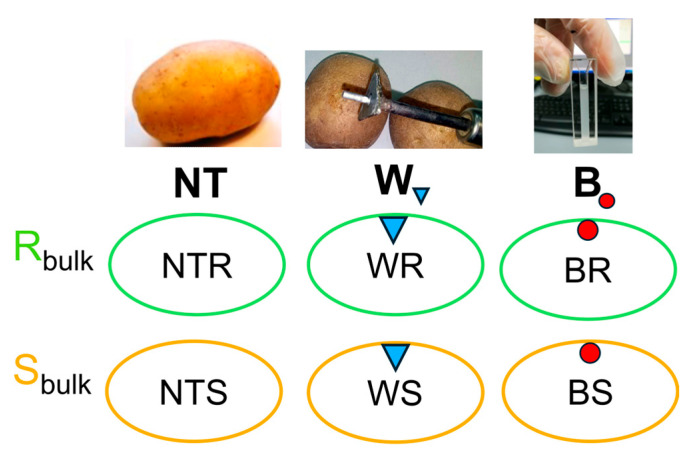
Experimental design illustrating treatment groups and sampling strategy. The schematic shows the three treatment conditions applied to resistant (R_bulk_, green) and susceptible (S_bulk_, orange) potato bulks. Each bulk was prepared from RNA extracted from tubers of five genotypes, with three tubers sampled from three different plants per genotype. Samples were collected at 8, 24, and 48 hours post-inoculation with *D. solani* for RNA-seq analysis. Treatments included NT (non-treated tubers), W (blue triangle—wounded tubers treated with sterile water), and B (red dot—wounded tubers inoculated with *D. solani*).

A graphical representation of the experimental design is shown in Figure 7. The experiment followed the methodology described by Lebecka et al. [9], with modifications for transcriptomic analysis. Tubers from five resistant (R_bulk_) and five susceptible (S_bulk_) genotypes of the DS-13 mapping population were divided into three treatment groups: non-treated (NT), wherein tubers were left uninjured and untreated; wounded (W), wherein tubers were punctured using a sterile steel rod (10 mm length, 2 mm diameter) and treated with 10 µL of sterile water; and a bacteria inoculated group (B), wherein tubers were wounded as in the W group and inoculated with 10 µL of *D. solani* suspension OD_600_ = 1.0 (10^9^ (CFU) mL^−1^). In total, 270 tubers were used in the experiment (10 genotypes × 3 tubers × 3 treatments × 3 time points). After treatment, tubers were placed in humid chambers, sprayed with sterile distilled water, and incubated at 27 °C in the dark to promote infection. Tissue samples (~250 mg per tuber) were collected from the inoculation sites using a cork borer at 8, 24, and 48 hours post-inoculation (hpi). The collected samples were immediately deep frozen in liquid nitrogen and stored at −80 °C for RNA extraction. For transcriptome analysis, 54 RNA samples were prepared, representing two genotype groups differing in resistance to *D. solani* (resistant and susceptible). For each treatment (*D. solani-inoculated*, wounded control, and untreated control), and each time point (8, 24, and 48 hpi), RNA was pooled from three biological replicates (three tubers from three plants per genotype). Each bulk represented RNA from five F_1_ genotypes selected for their extreme phenotypic response to *D. solani*.

The resistance of 176 potato genotypes to *D. solani* in potato tubers was assessed during the research, according to the method described by Lebecka et al. [9]. Assessments were carried out on tubers over three consecutive growing seasons. The results of ten selected for this study of potato genotypes (36 tubers in total: three tubers per genotype, three years × two dates × two replicates) were used for statistical analysis, together with the results of the test performed alongside the sequencing experiment. A total of 41 tubers from each genotype were evaluated. Statistical analyses were performed using Statistica 10 software (Statsoft Inc., Tulsa, OK, USA). The significance of differences among potato clones was estimated using Duncan’s test, followed by the analysis of variance (ANOVA). In addition, 5 tubers of each genotype (50 tubers in total) were inoculated alongside the tubers used for RNA-seq studies and incubated for three days to evaluate symptoms.

### 4.4. RNA Isolation and Preparation of Sequencing Samples

Total RNA was isolated using the Direct-zol RNA Miniprep kit (Zymo Research, Irvine, CA, USA) following the manufacturer’s protocol. The quality and quantity of the RNA was assessed using a NanoDrop Lite spectrophotometer (Thermo Fisher Scientific, Waltham, MA, USA). Sequencing samples were suspended in RNase-free, protein-free water. The quality criteria for RNA sequencing were as follows: RNA yield: ≥2 µg, volume: ≥20 µL concentration: ≥40 ng/µL, purity: OD_260/280_ = 2.0–2.2, RNA integrity: 28S:18S ratio ≥ 1.0; RIN ≥ 7.0

### 4.5. RNA Sequencing

RNA sequencing was performed using the NovaSeq 6000 platform (Illumina Inc., San Diego, CA, USA) in paired-end mode (2 × 150 bp reads). Libraries were prepared using the NEBNext^®^ Ultra™ II Directional RNA Library Prep Kit for Illumina^®^ (NEB) (New England Biolabs, Ipswich, MA, USA) by Genomed S.A (Warsaw, Poland).

### 4.6. Bioinformatics Analysis

The primary objective of the bioinformatics analysis was to compare gene expression profiles between samples and identify genes with statistically significant differences in expression. The workflow included the following steps: Adapter trimming: Adapter sequences were removed using Cutadapt [38]. Quality filtering: Reads were filtered using a quality threshold of Q ≥ 25 and a minimum read length of 15 bp. Quality assessment: FASTQC, available at https://www.bioinformatics.babraham.ac.uk/projects/fastqc/ (accessed on 25 August 2025), was used to generate quality control reports, which were summarised in a comprehensive quality assessment report. Read mapping: Filtered reads with a minimum length of 20 bp were mapped to the reference genome of *Solanum tuberosum* L. (NCBI accession: GCF_000226075.1_SolTub_3.0) using Hisat2 [39] with RNA-strand RF library preparation. Gene-level counts: Gene-level read counts were calculated using HTSeq [40] with the stranded reverse option (--stranded=reverse) to distinguish transcript strands. Gene annotation: Gene annotations were assigned using the feature_table.txt file from the *Solanum tuberosum* reference genome and the PlantRegMap database [41]. Differential gene expression analysis was performed in R v4.2.2, available at https://www.R-project.org/ using DESeq2 v1.38.3 [42], with FDR correction using the Benjamini–Hochberg method. A single-factor model was used for data of each time point separately. The complete list of genes tested in DESeq2 that passed the preliminary filtering stage (with a total of ≥3 counts across all samples) and had the appropriate annotation (GO/Entrez) was used to identify biological pathways. These pathways were determined using the KEGG database [43] and the clusterProfiler package v4.0 [44] for statistically significant genes (*p* ≤ 0.01) with a log_2_ fold change ≥2 and an assigned Entrez ID. Bioinformatics analysis was performed by Genomed S.A., Warszawa, Poland

### 4.7. Reverse Transcription and cDNA Synthesis

RNA (0.5 μg) extracted from the samples was subjected to reverse transcription (RT) to synthesise complementary DNA (cDNA) for downstream PCR and qPCR analyses. The reaction was performed using the TaqMan^TM^ MicroRNA Reverse Transcription Kit (Applied Biosystems, Foster City, CA, USA) following the manufacturer’s protocol. The reaction conditions included incubation at 16 °C for 30 min, followed by 42 °C for 30 min, and enzyme inactivation at 82 °C for 5 min.

### 4.8. PCR Amplification

The PCR reactions were set up using 2 × PCR mix Plus (A&A Biotechnology, Gdynia, Poland), 1 μL of primer mix (20 μM each), 1 μL of 5-fold diluted cDNA template, and nuclease-free water to the final volume. The thermal cycling conditions were as follows: initial denaturation at 95 °C for 3 min; 35 cycles of 95 °C for 10 s, annealing at 65 °C for 20 s, and elongation at 72 °C for 30 s; followed by a final extension at 72 °C for 10 min. PCR products were separated on a 2% agarose gel in TBE buffer and visualised under UV light after ethidium bromide staining, using DirectLoad™ PCR 100 bp Low Ladder (Invitrogen, Waltham, MA, USA) as a size marker. The PCR products were separated on 1.2% agarose gel prepared with Tris-borate-EDTA buffer, and fragment sizes were compared against a DNA ladder (Perfect 50 bp, 100 bp DNA Ladder (A&A Biotechnology, Gdańsk, Poland). Visualisation of amplicons was performed under UV light.

### 4.9. Quantitative PCR Analysis

To verify the quality of synthesised cDNA prior to downstream applications, semi-quantitative PCR (semi-PCR) was performed. Each 10 μL reaction contained 5 μL of 2 × PCR mix Plus (A&A Biotechnology, Poland), 1 μL of primer mix (20 μM each), 1 μL of 5-fold diluted cDNA template, and nuclease-free water to the final volume. The thermal cycling conditions were as follows: initial denaturation at 95 °C for 3 min; 35 cycles of 95 °C for 10 s, annealing at 62 °C, 65 °C, or 68 °C for 20 s, and elongation at 72 °C for 30 s; followed by a final extension at 72 °C for 10 min. PCR products were separated on a 2% agarose gel in TBE buffer and visualised under UV light after ethidium bromide staining, using DirectLoad™ PCR 100 bp Low Ladder (Invitrogen, Waltham, MA, USA) as a size marker. Candidate genes identified from the transcriptome analysis were subsequently evaluated using real-time quantitative PCR (qPCR). Reactions were performed in 10 μL volumes, containing 5 μL of SYBR Green PCR MasterMix (Thermo Fisher Scientific, Waltham, MA, USA), 1 μL of cDNA template, 1 μL of specific primer mix (20 μM each), and 3 μL of nuclease-free water. Each sample was analysed in four technical replicates, and no-template controls (NTCs) were included to ensure reaction specificity. Reactions were carried out on a LightCycler^®^ 480 system (Roche Diagnostics, Basel, Switzerland). The thermal cycling protocol was as follows: initial denaturation at 95 °C for 5 min; 40 cycles of denaturation at 95 °C for 10 s, annealing at 65 °C for 20 s, and extension at 72 °C for 30 s with fluorescence measurement; followed by a melting curve analysis with fluorescence detection at increments of 0.5 °C every 10 s starting from 68 °C. Relative gene expression levels were calculated using the 2^−ΔCt^ method, where ΔCt = Ct (target gene) − Ct (reference gene), as described previously [45]. Primer sequences, gene IDs, annealing temperatures, and reaction conditions are listed in Supplementary Table S1.

## 5. Conclusions

In this study, bulk segregant RNA sequencing (BSR-seq) analysis was utilised to show secondary metabolic pathways associated with tuber resistance to *D. solani* in diploid potato plants, as they are complex interspecific hybrids of *Solanum* species [9]. Among six SM pathways involved in the resistance, the category for the phenylpropanoid biosynthesis was the most abundant. Real-time PCR results for the expression of three genes associated with phenylpropanoid biosynthesis metabolism were highly consistent with those of bulk RNA-seq data, suggesting that changes in transcriptional profiling were reliable in identifying DEGs. Our findings suggest that the rapid activation of structural and immune-related pathways may contribute to the higher level of resistance in potato tubers. These data provide a basis for further studies of gene function and the molecular mechanism of potato resistance to *D. solani.* In particular, the suberisation-associated anionic peroxidase gene *POPA* (LOC102577694) was identified as a strong candidate contributing to tuber resistance at an early phase. However, the abundance of mRNA transcripts only partially correlates with protein abundances, and these relationships are complex [46]. In potato leaves, *POPA*-mediated oxidative burst in the apoplast was involved in JA-dependent signal triggering during defence against *Phytophthora infestans* [47]. Therefore, a gene knockout technique or overexpression studies might be used as the helpful tools for research on the gene *POPA* function in potato tubers. 

## Figures and Tables

**Table 1:** Number of genes in differentially expressed gene (DEG) analysis in BS vs. BR, WS vs. WR, and NTS vs. NTR at three time points, 8, 24, and 48 hours post-inoculation (hpi) with *D. solani*.

Time (hpi)	Treatment	Number of Genes				
		Total	Significant (*p* ≤ 0.01)			
			Upregulated		Downregulated	
				Log_2_ Fold Change ≥ 2		Log_2_ Fold Change ≤ −2
8	BS vs. BR	21,068	3134	566	3345	170
	WS vs. WR	21,228	2000	229	2457	140
	NTS vs. NTR	20,730	2768	443	2937	231
24	BS vs. BR	20,695	3339	822	3164	485
	WS vs. WR	21,196	3391	400	3606	544
	NTS vs. NTR	20,063	3857	858	3737	819
48	BS vs. BR	22,337	2879	675	2693	348
	WS vs. WR	22,105	2787	1014	2811	204
	NTS vs. NTR	21,520	3034	396	3253	861

Number of differentially expressed genes (DEGs) in resistant (R) vs. susceptible (S) potato genotype bulks at three time points (8, 24, and 48 hours post-inoculation, hpi) with *D. solani*. Comparisons include BS vs. BR (inoculated tubers), WS vs. WR (wounded control), and NTS vs. NTR (untreated control). Values are shown for total DEGs (*p* ≤ 0.01) and for significantly up- or downregulated genes (log_2_ fold change ≥ 2).

**Table 2:** Upregulated DEGs between WS vs. WR and BS vs. BR at 8, 24, and 48 hpi.

Gene ID	Log_2_ Fold Change						Functional Annotation
	WS vs. WR hpi			BS vs. BR hpi			
	8	24	48	8	24	48	
Phenylpropanoid biosynthesis							
102596330	4.65	-	7.41	4.62	-	6.88	Acetyl-CoA-benzyl alcohol acetyltransferase-like
102578320	-	-	-	4.49	-	-	acetyl-CoA-benzyl alcohol acetyltransferase-like
102599828	-	-	-	4.42	6.53	3.58	Lignin-forming anionic peroxidase-like
102598379	-	-	-	4.34	-	-	Salutaridinol 7-O-acetyltransferase-like
102591916	4.18	-	-	4.33	-	-	Peroxidase 43-like
102580092	3.76	2.03	-	4.21	-	-	Peroxidase P7-like
102605292	3.86	-	-	2.5	-	2.74	Suberisation-associated anionic peroxidase 2-like
102577694	-	-	-	2.87	3.99	5.35	Suberisation-associated anionic peroxidase, *POPA*
107063518	-	-	3.88	2.47	2.43	-	Peroxidase 21
102601606	2.02	-	-	2.64	-	5.69	Cationic peroxidase 1-like
102589793	-	-	-	2.43	3.72	-	4-coumarate-CoA ligase-like 6
102584603	-	-	-	2.39	3.56	2.95	4-coumarate-CoA ligase 2
102588483	-	-	-	2.06	2.61	-	4-coumarate-CoA ligase-like 9
102588050	-	-	2.12	4.96	-	-	Lignin-forming anionic peroxidase-like
102580211	-	-	-	2.29	3.77	-	Cinnamoyl-CoA reductase 1
102586332	2.93	-	-	3.61	6.03	-	Caffeoyl-CoA O-methyltransferase-like
Phenylpropanoid biosynthesis; Phenylalanine metabolism							
102582618^2^	2.06	2.35	-	4.05	4.36	4.04	Phenylalanine ammonia-lyase-like
Phenylalanine metabolism							
102591703	2.12	-	4.15	3.6	6.85	4.07	Histidine decarboxylase-like
102581954	3.38	-	-	3.34	5.27	2.00	
102592044	2.06	-	2.43	3.31	3.3	3.84	
102581292	-	-	-	3.29	3.45		
102580376	-	-	2.09	2.32	4.02	-	
102579686	-	-	-	2.07	2.29	-	Putative amidase
Phenylalanine metabolism; Tyrosine metabolism; Isoquinoline alkaloid biosynthesis							
102583802^3^	-	-	3.38	4.71	5.02	-	Primary amine oxidase-like
102600413^3^	-	-	4.16	4.03	2.52		
102581835^3^	2.07	-	-	2.43	2.16	2.07	
Tyrosine metabolism; Isoquinoline alkaloid biosynthesis							
102604380^2^	-	2.67	-	3.14	2.89		Polyphenol oxidase D, chloroplastic
102577727^2^	2.7	2.49	0.49	3.1	-	2.67	PPO, Catechol oxidase B
102597889	3.22	-	-	3.35	8.54		Alcohol dehydrogenase 1
102599478	2.33	-	2.52	2.48	-	2.58	
Amino sugar and nucleotide sugar metabolism							
102590097	-	3.09	-	5.59	5.18	2.39	Endochitinase 3-like
102590998	-	2.95	-	4.39	3.8	3.89	Basic endochitinase
102592970	-	-	-	2.64			*Solanum tuberosum* endochitinase 4-like
102583127	-	-	-	2.14	4.63	2.34	Endochitinase EP3
102577835	-	2.06	2.30	2.04	2.08		Acidic endochitinase pcht28
102584052	3.22	-	-	2.48	1.47	-	Alpha-1,4-glucan-protein synthase [UDP-forming] 2-like
102600109	-	-	1.7	2.25	-	2.22	Acidic mammalian chitinase-like
107059944	-	-	-	3.65	2.48	2.02	Chitotriosidase-1-like
102599450	-	-	2.07	3.33	2.96	2.3	
102594281	-	2.16	-	2.17	2.8		Mannose-1-phosphate Guanylyltransferase 1-like
Glutathione metabolism							
102599376	-	5.31	3.93	4.63	5.18	2.70	Probable glutathione S-transferase
102600356	2.49	-	2.03	2.61	4.96	3.15	
102599054	-	-	3.43	2.6	5.01	3.56	
102602051	-	-	4.48	2.41	2.23	5.17	
102604161	3.75	7.39	5.21	2.41	5.54	5.59	Glutathione S-transferase L1-like

**Each row represents DEGs with their respective gene ID and functional annotation.** Dashes (-) denote non-significant expression changes. Genes shared between pathways are marked with superscripts.

**Table 3:** DEGs between BS vs. BR at 8 hpi.

Gene ID	Log_2_ Fold Change	Physical Position on the Chromosome ^a^		Functional Annotation
		Beginning	End	
		ChrII		
LOC102589748	5.26	22771975	22769355	Probable N-acetyltransferase *HLS1*
LOC102590998	4.39	19537268	19539189	Basic endochitinase [*Solanum tuberosum* (potato)]
LOC102580833	4.21	33215044	33210712	NAC domain-containing protein 73 [*Solanum tuberosum* (potato)]
LOC102604927	4.04	41059989	41058531	*ST1* homolog
LOC102594583	3.88	29338193	29336454	Tetrahydrocannabinolic acid synthase-like
LOC102594665	3.54	36280755	36276180	Protein DETOXIFICATION 40-like
LOC102605761	3.02	17250771	17248588	*WRKY* transcription factor 6-like
**LOC102577694**	**2.87**	**35172274**	**35169042**	**Suberisation-associated anionic, *POPA***
LOC102582119	2.73	47475919	47479431	NAC domain-containing protein 8
LOC102579839	2.16	27067654	27064953	2-oxoglutarate-dependent dioxygenase
LOC102005497	2.13	33319425	33320828	Protein E6
LOC102579526	2.06	20712931	20711738	*GATA* transcription factor 5-like
LOC102583510	2.05	26944018	26946758	UDP-glycosyltransferase *87A2*-like
LOC102578310	2.03	35301108	35305753	G-type lectin S-receptor-like Serine/threonine-protein kinase *At1g11300*
		ChrIV		
LOC102603519	4.40	49272319	49274102	Premnaspirodiene oxygenase-like
LOC102602839	3.31	49258137	49260195	Cytochrome P450 *71D7*
LOC102582168	2.55	52289066	52291659	L-ascorbate oxidase-like
LOC102601591	2.19	52050946	52058860	General transcription factor IIH subunit 2

^a^ Positions in the reference genome *S. tuberosum* Group Phureja DM1-3 (v. 4.04, Hardigan et al. [18]) defined by BLAST+ v2.12.0 (NCBI) search results. Bold—the gene identified in enriched KEGG pathways (Table 2).

## Data Availability

The data presented in this study are available in the article and Supplementary Materials.

## Supplementary Materials

The supporting information can be downloaded at: https://www.mdpi.com/article/10.3390/ijms26178370/s1.
