# Supplementary material for: New Insights into the Role of Secondary Metabolic Pathways in Resistance of Potato to Dickeya solani
Source: Int J Mol Sci. 2025 Aug 28;26(17):8370. doi: 10.3390/ijms26178370 (PMC12429155; doi:10.3390/ijms26178370)
Supplement: Supplementary file 1 [file ijms-26-08370-s001.zip › ijms-3800862-supplementary.pdf]

Supplementary Table S1. Gene accession numbers, primer sequences, and product lengths for RT-qPCR analyses.

Genes	Gene ID	Forward Primer	Reverse Primer	Temp of Primer Annealing (°C)	Concentration in Reaction (μM)	Product Length (bp)
**Lignin-forming anionic peroxidase-like**	LOC102599828	TGGCTGCATCCCTCATTCGT	TGGCAATGCAGTCTTCTCGC	65	2,0	113
**Acidic endochitinase pcht28**	LOC102577835	CTGGTGATGATACCGCCCGT	ACCTTGTCCAGCTCGTTCGT	62	1,0	197
**Basic endochitinase**	LOC102590998	ACTACTGGTGGATGGGCAAC	TATTGCTCTCCCTGCTGGTC	65	1,2	195
**UDP-glycosyltransferase 87A2-like**	LOC102583510	GCTGCCCAACACAACCTTCG	TGTGGCCTCTACCTGGATATGG	65	1,2	163
**Cytochrome P450 71D7**	LOC102602839	CTCCCTACTGGAATGGAGCC	GGTAACTGTCACGCGGATACA	65	1,2	191
**Chitotriosidase-1-like**	LOC107059944	TCACGAGCACAGTTATGGGG	GGTGTTCTCGCCATGACTCCA	65	1,2	103
**Suberization-associated anionic peroxidase**	LOC102577694	TGCGTTTTCTCAGCTGTACG	GGTGTGAGTTTTGTTCCCC	65	1.0	173
**Peroxidase 21**	LOC107063518	CGGTGGACCCGACTATCGAC	GTCAGGGTTTGGTGACGTGC	65	1.0	71
**Peroxidase 43-like**	LOC102591916	GTTGCTGCCTCCAGCCAAAA	ACCCATCACACCCCTGAACG	65	1.0	82
ß-Tubulin		GTTGGCAATTCAACCTCCAT	ATGTTGCTCTCGGCTTCAGT	65	1.0	(Nahar et al., 2017)
